# Mixed-methods process evaluation of ctDNA use to guide decision-making in patients with advanced solid cancers: study protocol for a substudy of the LIQPLAT trial

**DOI:** 10.1136/bmjopen-2025-100537

**Published:** 2025-10-28

**Authors:** Johannes M Schwenke, Andreas M Schmitt, Stuart McLennan, Perrine Janiaud, Heinz Läubli, Mascha Binder, Ilaria Alborelli, Matthias S Matter, Jennifer Hinke, Corinne C Widmer, Lars G Hemkens, Benjamin Kasenda, Matthias Briel

**Affiliations:** 1 CLEAR Methods Center, Division of Clinical Epidemiology, Department of Clinical Research, University Hospital Basel, Basel, Switzerland; 2 Division of Medical Oncology, University Hospital Basel, Basel, Switzerland; 3 Institute of History and Ethics in Medicine, Department of Preclinical Medicine, TUM School of Medicine and Health, Technical University of Munich, Munich, Germany; 4 Department of Clinical Research, University of Bern, Bern, Switzerland; 5 Pragmatic Evidence Lab, Research Center for Clinical Neuroimmunology and Neuroscience Basel (RC2NB), University Hospital Basel, Basel, Switzerland; 6 Meta-Research Innovation Center, Stanford University, Stanford, California, USA; 7 Laboratory of Translational Immuno-Oncology, Department of Biomedicine, University Hospital Basel, Basel, Switzerland; 8 Department of Pathology, University Hospital Basel, Basel, Switzerland; 9 Division of Hematology, University Hospital Basel, Basel, Switzerland

**Keywords:** Clinical Protocols, Molecular aspects, Implementation Science, ONCOLOGY, Clinical Trial

## Abstract

**Introduction:**

There is an urgent need to better understand how information from circulating tumour DNA (ctDNA) can be integrated into routine care for patients with advanced solid cancer.

**Methods and analysis:**

The implementation of liquid biopsies in routine care of patients with advanced solid cancer trial (LIQPLAT) is a single-centre, single-arm trial investigating the implementation of ctDNA in the routine care of patients with advanced solid cancer. We present a mixed-methods process evaluation embedded in the LIQPLAT trial, following Medical Research Council guidance and the Reach, Effectiveness, Adoption, Implementation, Maintenance framework. We show a logic model, which details the causal chain and related assumptions from recruiting patients into the trial to the goal of improving quality of life and survival. Data collection is longitudinal and includes: semistructured interviews with healthcare professionals (pathologists, biologists, oncologists; planned n=20) and patients (planned n=15) to identify implementation barriers and facilitators; recordings of molecular tumour board meetings to analyse clinical decision-making; the 23-item Normalisation MeAsure Development survey for healthcare professionals (planned n=20) at four time points. Quantitative data from hospital records will be used to assess implementation outcomes like patient acceptance rates and ctDNA workflow success. Qualitative data will undergo thematic and content analysis, and quantitative data will be analysed using a Bayesian framework.

**Ethics and dissemination:**

The LIQPLAT trial was approved by the regional ethics committee of Northwestern and Central Switzerland (BASEC 2024-00358). The qualitative aspects of the process evaluation were exempted from ethics review according to the Swiss Human Research Act. We follow guidelines for data security, confidentiality and information governance. Results will be submitted for publication in peer-reviewed journals and discussed at conferences.

**Trial registration number:**

NCT06367751, SNCTP000005844.

**STRENGTHS AND LIMITATIONS OF THIS STUDY**

- Integration of interviews with patients and healthcare professionals, repeated questionnaires, clinical data and recorded molecular tumour board (MTB) meetings enables tracking routine circulating tumour DNA (ctDNA) measurement implementation and impact from laboratory analysis to clinical decisions.
- Video recordings of MTB meetings over 30 months allow for direct observation of clinical decision-making processes based on ctDNA and their evolution, rather than relying on retrospective accounts.
- As a single-centre study, findings may have uncertain generalisability to other healthcare settings, particularly those with different resource levels or organisational structures.

## Introduction

 Liquid biopsies, including analysis of circulating cell-free tumour DNA (ctDNA), may allow for better personalisation of cancer treatment,^1^,^4^ but implementing this technology in routine clinical practice presents significant challenges. The path from blood sampling to clinical decision-making is complex, involving challenges in laboratory processing,^5^ expert interpretation^6^ and integration into existing clinical workflows. Calls for wider availability of ctDNA in routine care are increasing,^7^ but studies on the implementation of ctDNA in routine care are scarce.

In this protocol, we outline a mixed-methods process evaluation embedded in the implementation of liquid biopsies in routine care of patients with advanced solid cancer trial (LIQPLAT), which investigates the effects of routine ctDNA measurements. Process evaluations enhance our understanding of why interventions succeed or fail, by examining factors such as intervention delivery, uptake and contextual factors.^8 9^ Integrating process evaluations with clinical trials can improve the understanding of causal pathways leading to effectiveness or ineffectiveness of an intervention and can aid stakeholders in interpreting the trial results.^9^

While the LIQPLAT trial will provide preliminary effectiveness data, the process evaluation aims to illuminate the steps from blood sampling to impact on patients’ health and well-being. These steps include the ctDNA sample analysis, interpretation of results, effective recommendations for clinical management by specialists, and adherence to these recommendations in actual patient care. By conducting a process evaluation, we aim to contextualise implementation challenges of routine ctDNA measurements, evaluate our assumptions about causal mechanisms and examine stakeholder attitudes and experiences.

### The LIQPLAT trial

The subject of this process evaluation is the LIQPLAT trial, a single-centre, single-arm trial designed to assess the feasibility of implementing routine ctDNA measurements for patients with advanced solid cancers and which includes valid comparisons with a registry-based control group. Conducted at the University Hospital of Basel in Switzerland, the trial enrols participants diagnosed with any type of advanced solid cancer, excluding primary brain tumours. The target enrolment is at least 150 participants who accept the intervention offer, with at least 75 registry-based concurrent controls. Detailed information on trial design and rationale can be found in the trial protocol^10^ and trial registry record (ClinicalTrials.gov, NCT06367751).

The intervention comprises repeated measurements of ctDNA which may trigger changes in clinical management alongside standard of care diagnostics and treatment. Measurements are scheduled at baseline, between 2–3 months and 5–7 months after treatment initiation, and on confirmed or suspected disease progression. Analysis of ctDNA will be performed by specialists at the university hospital’s department of pathology using either the Oncomine Pan-Cancer Cell-Free Assay^11^ or a custom prostate cancer panel,^12^ both from Thermo Fisher Scientific. Results are reviewed and discussed at an interdisciplinary molecular tumour board (MTB). The MTB has knowledge of the oncological disease history of the respective patients. The recommendations of the MTB are summarised in a report for the clinical team which is part of the electronic health record.

The MTB plays a pivotal role in translating ctDNA analysis results into actionable clinical decisions. Due to the heterogeneity of diseases and associated treatment histories, predefined, rigid rules governing the MTBs recommendations based on ctDNA results were not defined a priori. Consequently, a core focus of this process evaluation is to examine how the MTB integrates ctDNA findings into their decision-making process.

### Aims and objectives

The overarching goal of this process evaluation is to gain a comprehensive understanding of how routine measurements of ctDNA are implemented within routine care and how they influenced clinical decision-making. The specific objectives are:

1. To examine the acceptance of the offer of routine ctDNA measurements by patients with advanced solid cancers.
2. To investigate the implementation of routine ctDNA measurements in clinical practice, that is, if ctDNA was sampled, analysed and made available to the tumour board as planned, and to identify associated barriers and facilitators.
3. To examine how the additional information from ctDNA measurements affected recommendations by the MTB.
4. To explore attitudes of stakeholders (patients, pathologists, biologists, oncologists) towards routine ctDNA measurements.
5. To assess when and why ctDNA measurements lead to an earlier termination of ineffective therapies, an increased delivery in targeted therapies, and whether this was associated with a reduction in treatment-associated side effects.

## Methods and analysis

### Design

This process evaluation uses a mixed-methods approach, focusing on implementation process factors as well as outcomes using quantitative and qualitative analyses. The evaluation is based on the RE-AIM (Reach, Effectiveness, Adoption, Implementation, Maintenance) framework,^13^ with adaptations where needed to tailor the evaluation to the LIQPLAT trial. The RE-AIM framework offers a systematic approach to evaluating how an intervention plays out in a real-world setting. For LIQPLAT, we defined reach to refer to the number and proportion of patients willing to participate in the trial and to take up the intervention, as well as their motivations for doing or not doing so. In this process evaluation, effectiveness will not refer to the effectiveness outcomes of LIQPLAT, that is, improvement in patient-reported quality of life and survival, as this is the subject of the LIQPLAT trial. However, it refers to proposed causal mechanisms potentially leading to improved quality of life, such as increased prescription of targeted therapies and reduction of treatment-associated side effects such as anaemia or thrombocytopenia requiring transfusion. For adoption*,* we will evaluate whether eligible patients were invited to the LIQPLAT trial as intended and whether the results of the ctDNA analysis were systematically considered by the MTB. For implementation, we will examine to which extent ctDNA was sampled, analysed and made available to the MTB as intended, as well as barriers and facilitators for its use, whether and how results from ctDNA analysis influenced recommendations of the MTB, and whether recommendations were followed in patient care and if not, why not. For maintenance*,* we will examine changes in the implementation over time and stakeholder attitudes towards and perceptions of more widespread adoption of ctDNA measurements.

### Logic model

**Figure 1:**
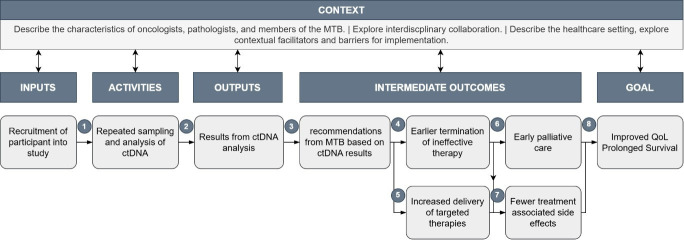
Logic model. The model shows the causal chain from recruiting a patient into the LIQPLAT trial to the goal, a relative improvement in quality of life and/or survival. The assumptions, evidence and rationale, as well as the planned evaluation for each numbered link are detailed in online supplemental table 1. ctDNA, circulating tumour DNA; MTB, molecular tumour board; QoL, quality of life.

In accordance with the UK Medical Research Council guidance,^9^ we developed a logic model for the LIQPLAT process evaluation through discussion with members of the MTB, patient representatives, practising oncologists, and our research group. The logic model (figure 1) was developed to clearly illustrate the theory of change, that is, causal chain of steps leading to the goal of improved quality of life and prolonged survival of patients. The selection of quantitative and qualitative data to collect, as well as the design of semistructured interviews, was derived from this logic model. The evaluation of each causal link was mapped to the corresponding component of RE-AIM (online supplemental table 1).

### Context

We anticipate a successful implementation of routine ctDNA measurements to be dependent on various contextual factors within the healthcare setting. We will explore resource availability, that is, type and availability of specialised equipment required for ctDNA analysis, the qualification and expertise of personnel involved in sample processing and results interpretation, as well as considerations related to cost. In the semistructured interviews with stakeholders, we will explore contextual barriers and facilitators, such as staffing shortages and interdisciplinary collaboration.

### Data collection

#### Individual interviews

From the logic model, we identified four key stakeholder groups for semi-structured interviews. Patients invited to the LIQPLAT trial, the pathologists and analysts carrying out the ctDNA analysis, members of the MTB and treating oncologists. We will develop semistructured interview guides for each stakeholder group (online supplemental appendix A), based on the assumptions of the logic model and the aims of the evaluation (online supplemental table 1). The interview guides will be discussed within the research group and with patient representatives. Interviews will be carried out by JMS (a male, early career researcher), in the language of preference of the interviewee (German, English or French). Interview guides will be piloted in the first two interviews for each stakeholder group and adapted where needed. Before the interviews, we will briefly explain the key aspects and motivation for the interview with each interviewee. We will ask all interviewees for permission to record the interview on an audio device and gather oral informed consent.

Interviews with patients will be carried out in person during the visit following the baseline consultation, which is usually a visit for the first dose of treatment. We decided against interviewing patients directly after their first contact with the oncology department, as this is usually a long and psychologically burdensome visit, and we assumed an additional interview to be an additional burden for the patient. The interview explores their reasons for accepting or declining the invitation, how they experienced the offer of ctDNA measurements, and their hopes and concerns. As we anticipate high acceptance of the invitation (~95%), we aim to interview most, if not all, patients who did not wish routine ctDNA measurements, though some may decline the interview. For a target sample size of 150 patients who accept the intervention, we thus expect around 8 patients to decline the intervention. Of the patients who accept the intervention, we will conduct an interview with a purposive sample of about 10%, that is, around 15 patients.

All interviews with members of the pathology department, members of the MTB and treating oncologists will be carried out in person, through phone or in a Microsoft Teams call. We plan two interview rounds, once before or shortly after the start of recruitment, and once after 12 months of recruitment. The interview guides for the second round will be developed and adapted based on the results of the first round of interviews.

We plan to interview most members of the pathology department directly involved in the analysis of ctDNA. The first round will explore expected challenges related to the analysis of an increased volume of ctDNA, communication between pathologists and oncologists, as well as attitudes towards utility and scalability of ctDNA analysis.

We plan to interview all members of the MTB. The initial interview will explore the attitudes of members of the MTB towards routine ctDNA measurements as well as the expected influence on their decision-making. The second round of interviews will focus on reflecting on the use of the additional information from ctDNA analysis in their decision-making process, and the perceived utility of ctDNA.

We also plan to interview oncologists, that is, residents, attending physicians and senior consultants on their views on the LIQPLAT trial, ctDNA and the recommendations received from the MTB. In total, we expect to interview approximately 20 healthcare professionals.

Interviews are transcribed using a locally hosted instance of Whisper.^14^ The transcripts will be checked for accuracy against the sound files and corrected by research assistants who have signed a confidentiality agreement.

#### Recordings of MTB meetings

Interdisciplinary MTB meetings take place every 14 days, on a Microsoft Teams call. A premeeting, during which the members of the MTB discuss upcoming cases and make preliminary decisions to be finalised at the biweekly meeting, takes place every 7 days, also on Microsoft Teams. Parts of the meetings, where a patient of the LIQPLAT trial is discussed, will be recorded using the recording function of Microsoft Teams (video and audio). Transcription and quality control will be performed as described above for the interviews. JMS will have the opportunity to attend the MTB meetings and to regularly approach members of the MTB for any unclarity about the decision-making process which arises during the analysis of the transcripts.

#### Normalisation MeAsure Development questionnaire

We will use the Normalisation MeAsure Development (NoMAD) instrument^15^ to assess the implementation of ctDNA measurements from healthcare professionals’ perspective. The NoMAD instrument consists of 23 items across four constructs: coherence (sense-making), cognitive participation (engagement), collective action (work done to enable the intervention to happen), and reflexive monitoring (appraisal). All involved healthcare professionals will be invited to complete the NoMAD questionnaire around 6, 12, 18 and 24 months after the beginning of the study. We will not conduct a preimplementation survey since this would require rewording the NoMAD items from experiential to anticipatory language. This would make longitudinal comparisons methodologically unsound as the underlying constructs would likely differ. The questionnaire will be administered electronically using REDCap. We adapted the generic NoMAD items to specifically reference ctDNA testing while maintaining the validated structure of the instrument. The NoMAD assessment will specifically strengthen our evaluation of the adoption, implementation and maintenance domains of RE-AIM. This structured assessment of normalisation processes will complement our qualitative data collection methods and help identify barriers and facilitators to sustained implementation.

#### Quantitative data collection

We will use routinely collected data from the hospital data warehouse for quantitative aspects of the process evaluation, as well as data from REDCap databases used for the LIQPLAT trial. Password protection and user-rights management ensure that only authorised personnel have access to the data.

#### Qualitative analyses

##### Interviews

For qualitative data analysis of the interviews, we follow an inductive approach.^16^ Using the interview transcriptions in their original language, conventional content analysis will be performed with the assistance of the qualitative software MAXQDA. Initial themes will be identified and labelled using a process of open coding. The findings will be presented as higher and lower-level categories.

##### Recordings of MTB meetings

The recordings, transcripts and reports of MTB meetings will be analysed to answer the following questions: (1) Did the MTB make a recommendation based on information from ctDNA? (2) What recommendation did the MTB make? (3) Did the recommendation pertain to treatment or diagnostics? (4) How did the members of the MTB justify the recommendation based on ctDNA? (5) Did the members of the MTB encounter any difficulties in operationalising ctDNA results for making clinical recommendations?

After analysis of a sample of MTB transcripts and recordings, we will refine questions in an iterative process in the research team and create an extraction template to be applied to all transcripts and recordings until saturation has been reached.

The findings from the analysis of MTB meetings will be triangulated with findings from interviews with MTB members.

### Quantitative analyses

We will use a Bayesian framework to quantify uncertainty for all estimates, presenting results as posterior medians with 95% credible intervals. To quantify process indicators and explore the intervention’s potential impact (see online supplemental table 1), we will use Bayesian regression models, including logistic, negative binomial and survival models as appropriate. This will allow us to estimate key metrics such as patient eligibility and acceptance rates, as well as the technical success and detection rates of the ctDNA workflow. Additionally, we will compare delivery of targeted therapies, time to treatment modification and blood product transfusion rates between the intervention group and a registry-based external control group.

For the NoMAD questionnaires, we will calculate descriptive statistics for each construct at each time point and visualise changes in scores over time. A detailed, version-controlled statistical analysis plan is publicly available on GitHub.^17^

### Patient and public involvement

We collaborated with two patient representatives to develop the study protocol and interview guides. Their input helped ensure the research questions and methods were patient centred. There are no plans to disseminate the results of the process evaluation directly to the interviewed patients.

### Ethics and dissemination

The regional ethics committee of Northwestern and Central Switzerland (EKNZ) reviewed and approved the protocol of the LIQPLAT trial (BASEC 2024-00358). The qualitative aspects of the process evaluation were exempted from the ethics review by the EKNZ according to the Swiss Human Research Act, if they did not directly concern human disease or structure and function of the human body. The participation of members of the different stakeholder groups in the semistructured interviews is voluntary. Before each interview, we will ensure that oral informed consent is obtained from each participant. Patients are additionally required to have signed the General Research Consent of the University Hospital Basel, as this is an eligibility criterion for the LIQPLAT trial. Since we did not seek permission from the EKNZ to make the interview data (transcripts) available on a data-sharing platform, the original data cannot be made publicly available but will be shared on a case-by-case basis.

### Confidentiality and coding

To protect participant confidentiality while enabling data linkage of patient data for robust analysis, a pseudonymisation process is applied to all collected data. Identifiable personal information of patients will be replaced with the unique patient hospital identification number, which will be used solely for internal data linkage. This allows for the triangulation of qualitative interview data and MTB data with quantitative clinical data.

### Code sharing

All code used for the quantitative analysis will be shared on a GitHub repository.

### Results dissemination

All results will be published in a peer-reviewed biomedical journal, whenever possible under open access, and discussed at conferences.

## Discussion

This protocol describes a comprehensive process evaluation designed to examine the implementation of routine ctDNA measurements in clinical practice alongside the LIQPLAT trial at a Swiss university hospital. To our knowledge, this is the first process evaluation examining the implementation of ctDNA in routine care specifically in the advanced cancer setting. A similar process evaluation is currently underway for a large trial in the adjuvant setting.^18^ Our approach has several strengths: first, the multi-stakeholder approach, encompassing patients, pathologists, MTB members and treating oncologists, ensures we capture the full spectrum of experiences and challenges across the implementation pathway.

This protocol describes a comprehensive process evaluation designed to examine the implementation of routine ctDNA measurements in clinical practice alongside the LIQPLAT trial at a Swiss university hospital. To our knowledge, this is the first process evaluation examining the implementation of ctDNA in routine care specifically in the advanced cancer setting. A similar process evaluation is currently underway for a large trial in the adjuvant setting.^18^ Our approach has several strengths: first, the multistakeholder approach, encompassing patients, pathologists, MTB members and treating oncologists, ensures we capture the full spectrum of experiences and challenges across the implementation pathway.

An important limitation of our evaluation is its single-centre design, which may limit generalisability to other healthcare settings, particularly those with different organisational structures or resource levels. However, many of the insights gained, especially regarding the interpretation and clinical integration of ctDNA results, should be broadly applicable across settings, as the fundamental challenges of result interpretation and clinical decision-making are likely to be similar across healthcare systems. An additional limitation is forgoing reinterviews with participants of the LIQPLAT trial during follow-up. We decided against this to limit Hawthorne effects, which could influence the comparison of patient-reported outcomes of LIQPLAT participants with the registry-based control group. The disadvantage of this approach is the resulting lack of data on patients’ understanding of and attitudes towards the results from ctDNA measurements in their care.

We anticipate that this process evaluation will provide valuable insights into the practical challenges and opportunities of implementing routine ctDNA measurements in clinical practice.

### Trial status

The first patient was randomly selected for invitation to the trial on 29 April 2024; the first patient was invited on 2 May 2024. As of 24 September 2025, 160 patients have been selected for invitation to the LIQPLAT trial, of which 132 participants have accepted the invitation. Recruitment is scheduled to be completed in October 2025. The first round of interviews with healthcare professionals and the first round of the NoMAD survey has been completed. Patient interviews, the second round of interviews with healthcare professionals and the second round of the NoMAD survey are ongoing.

## Supplementary material

10.1136/bmjopen-2025-100537 online supplemental file 1

10.1136/bmjopen-2025-100537 online supplemental file 2
