# Supplementary material for: Mixed-methods process evaluation of ctDNA use to guide decision-making in patients with advanced solid cancers: study protocol for a substudy of the LIQPLAT trial
Source: BMJ Open. 2025 Oct 28;15(10):e100537. doi: 10.1136/bmjopen-2025-100537 (PMC12570924; doi:10.1136/bmjopen-2025-100537)
Supplement: online supplemental file 1 [file bmjopen-15-10-s001.docx]

# Supplementary Table 1

| **Process evaluation details according to the logic model, and related RE-AIM constructs.**  **Abbreviations: CHIP, clonal hematopoiesis of indeterminate potential;** ctDNA, circulating tumor DNA; MTB, molecular tumor board; QoL, quality of life. | | | | |
| --- | --- | --- | --- | --- |
|  | **Assumptions** | **Prior evidence / rationale** | **Planned evaluation** | **RE-AIM constructs** |
| **1)** | - Enough patients will be eligible to receive ctDNA measurements. - Enough patients will accept the invitation to receive repeated ctDNA measurements as part of the LIQPLAT trial. | The intervention only places a minimal, if any, burden on trial participants. The liquid biopsy will take place at the same time as routine blood draws, thus avoiding an additional venipuncture and patient discomfort. | Quantitative:   - Number and proportion of patients who are in principle eligible for LIQPLAT trial. - Number and proportion of patients who are scheduled to be invited to the trial and are in fact offered the invited by their physician. - Number and proportion of patients who accept the invitation.   Qualitative:   - Semi-structured interviews with all patients who declined the invitation, and a purposive sample of patients who accepted the invitation. For the interview guides see *Supplementary Appendix A*. | Reach, Adoption |
| **2)** | - Patients who have agreed to routine measurements of ctDNA analyses receive these analyses as scheduled. - The blood samples arrive in a processable state in the department of pathology. - The analyses carried out in the department of pathology have high sensitivity and specificity, are standardized, and have a low technical error rate. - CHIP does not pose a major challenge for the interpretation of results. - CtDNA is detectable in a sufficient proportion of patients. | - The study team will consist of a study coordinator, who will be responsible for supervising that ctDNA analyses have been correctly scheduled. - The blood for ctDNA analyses will be drawn using Streck Tubes, which limit lysis of white blood cells [1]. - The department of pathology has standardized operating procedures in place for the analysis of ctDNA, and already routinely analyzed a limited amount of ctDNA. - For solid tumors, Oncomine™ Pan-Cancer Cell-Free Assay will be used, which has a sensitivity of 80% at a variant allele frequency of 0.1%, with a specificity of 98%. | Quantitative:   - Proportion of ctDNA samples analyzed without error. - Proportion of ctDNA-analyses detecting ctDNA at baseline. - Proportion of variants found on ctDNA-analyses being labelled with suspicion of CHIP. - NoMAD questionnaire at 6m, 12m, 18m, 24m   Qualitative   - Semi-structured interviews with pathologists carrying out the analysis. For the interview guide see *Supplementary Appendix A*. - Triangulation of NoMAD results with interview data | Implementation, Maintenance |
| **3)** | - Results from the ctDNA analysis will be used by the MTB to reach a counterfactually different recommendation than they would have without the information on ctDNA. | - To the best of our knowledge, this topic has not been previously studied. Our research provides the initial evidence. | Qualitative:   - Semi-structured interviews with the members of the MTB shortly after start of recruitment and after 1 year. See *Supplementary Appendix A* for the interview guides. - We will record all sessions of the MTB concerning participants enrolled in LIQPLAT. | Implementation |
| **4)** | - Analysis of variant allele frequency and its dynamics allow for correct identification of ineffective therapies. - The MTB will be able to accurately extract this information from the results of the ctDNA analysis and recommend the stop of ineffective therapies earlier, than in a counterfactual without results from ctDNA analysis. - The recommendations from the tumor board will be executed by the treating oncologists | - Observational data suggests that ctDNA baseline data and kinetics predict response to treatment [2–4]. | Quantitative:   - Comparison of time from treatment initiation to treatment switch or stop (composite) with external control. - NoMAD questionnaire at 6m, 12m, 18m, 24m   Qualitative:   - We will analyze the recordings of the sessions of the MTB as well as the written reports from the MTB to evaluate whether the members of the MTB recommended treatment stop for ineffectiveness based on results from ctDNA. - Semi-structured interviews with treating oncologists - Triangulation of NoMAD results with interview data | Implementation, Effectiveness |
| **5)** | - The ctDNA analyses allow for the identification of more targets for targeted therapy. - The MTB will recommend a switch to more targeted treatments. - The recommendations from the tumor board will be executed by the treating oncologist. | - In patients with NSCLC ctDNA lead to an increase of the detection of targetable mutations and increased delivery of molecularly guided therapy [5]. | Quantitative:   - Number and proportion of patients with actionable alterations in ctDNA analysis (ESCAT grade 1 or 2). - Comparison of proportion of patients receiving targeted therapies between intervention and external control. - NoMAD questionnaire at 6m, 12m, 18m, 24m   Qualitative:   - We will analyze the recordings of the sessions of the MTB as well as the written reports from the MTB to evaluate whether the members of the MTB recommend a targeted therapy based on results from ctDNA. - Semi-structured interviews with treating oncologists - Triangulation of NoMAD results with interview data | Implementation, Effectiveness |
| **6)** | - Earlier termination of ineffective therapy will lead to fewer treatment associated side effects. - Earlier termination of ineffective therapy will lead to early palliative care. | - Earlier termination of ineffective therapy logically entails the reduction of treatment associated side effects. - In case of treatment failure without alternative, patients will receive palliative care, whose explicit goal is to alleviate symptoms | Quantitative:   - Number of blood products (red blood cell transfusions and platelet transfusions) per patient. - Time to switch from first-line treatment to palliative care | Effectiveness |
| **7)** | - Increased delivery of targeted therapy will reduce treatment associated side-effects compared to first-line systemic therapy. | - Targeted therapies are generally considered as having fewer side-effects, compared to first-line systemic therapy [6,7]. | Quantitative:   - Proportion of patients receiving targeted therapy - Proportion of participants recruited into other studies | Effectiveness |
| **8)** | - Earlier palliative care and fewer treatment associated side effects will improve patients’ QoL. - Patients’ QoL can be accurately assessed using a questionnaire. - Patients are offered and reliably fill out the questionnaire | - Early palliative care in metastatic NSCLC significantly improves QoL and might prolong survival [8]. - Early palliative care in advanced cancer improved QoL on four scales 4 months after baseline [9]. - Routine collection of QoL data is already established for all cancer patients, and we have observed response rates of about 90%. | Quantitative:   - Median number of QoL questionnaires filled by patients in 6 months and in 12 months. - Proportion of returned incomplete questionnaires. - Proportion of pats with baseline QoL only (no follow-up questionnaires filled) | Effectiveness, implementation |

## Bibliography

1 Dang DK, Park BH. Circulating tumor DNA: current challenges for clinical utility. *J Clin Invest*. 2022;132. doi: 10.1172/JCI154941

2 Bratman SV, Yang SYC, Iafolla MAJ, *et al.* Personalized circulating tumor DNA analysis as a predictive biomarker in solid tumor patients treated with pembrolizumab. *Nat Cancer*. 2020;1:873–81.

3 Keller L, Guibert N, Casanova A, *et al.* Early Circulating Tumour DNA Variations Predict Tumour Response in Melanoma Patients Treated with Immunotherapy. *Acta Derm Venereol*. 2019;99:206–10.

4 Sivapalan L, Murray JC, Canzoniero JV, *et al.* Liquid biopsy approaches to capture tumor evolution and clinical outcomes during cancer immunotherapy. *J Immunother Cancer*. 2023;11. doi: 10.1136/jitc-2022-005924

5 Aggarwal C, Thompson JC, Black TA, *et al.* Clinical Implications of Plasma-Based Genotyping With the Delivery of Personalized Therapy in Metastatic Non-Small Cell Lung Cancer. *JAMA Oncol*. 2019;5:173–80.

6 Basak D, Arrighi S, Darwiche Y, *et al.* Comparison of Anticancer Drug Toxicities: Paradigm Shift in Adverse Effect Profile. *Life*. 2021;12. doi: 10.3390/life12010048

7 Kroschinsky F, Stölzel F, von Bonin S, *et al.* New drugs, new toxicities: severe side effects of modern targeted and immunotherapy of cancer and their management. *Crit Care*. 2017;21:89.

8 Temel JS, Greer JA, Muzikansky A, *et al.* Early palliative care for patients with metastatic non-small-cell lung cancer. *N Engl J Med*. 2010;363:733–42.

9 Zimmermann C, Swami N, Krzyzanowska M, *et al.* Early palliative care for patients with advanced cancer: a cluster-randomised controlled trial. *Lancet*. 2014;383:1721–30.
