# Supplementary material for: Mixed-methods process evaluation of ctDNA use to guide decision-making in patients with advanced solid cancers: study protocol for a substudy of the LIQPLAT trial
Source: BMJ Open. 2025 Oct 28;15(10):e100537. doi: 10.1136/bmjopen-2025-100537 (PMC12570924; doi:10.1136/bmjopen-2025-100537)
Supplement: online supplemental file 2 [file bmjopen-15-10-s002.docx]

# Appendix A

### Interview guide: Treating physicians (Baseline)

#### Introduction

Thank you for taking a few minutes to speak with me.

As described in the email, we would like to explore your expectations and opinions about the routine measurements of ctDNA.

I would like to ask you about your expectations regarding the impact of ctDNA on patient care.

If you agree, I would like to record our conversation, so I do not need to take notes. The transcripts will be anonymized so that you cannot be identified.

Do you have any questions before I start recording our conversation?

#### Questions

How do you rate the potential of ctDNA analysis to improve patient care and patient-relevant outcomes?

What are your hopes?

What are your concerns?

Do you expect that the treatment of your patients will change due to the routine measurements of ctDNA?

If yes, how?

At what point in the disease progression do you expect an impact from ctDNA?

If no, why not?

Do you anticipate differences in the utility of ctDNA among different patient groups / cancer diagnoses?

If yes, which groups do you expect to benefit the most?

For which groups do you foresee lesser or no benefit?

LIQPLAT is a study that is fully embedded in the routine care of patients. What is your opinion on this?

Do you have concerns, e.g., because of the workload for you personally?

How do you assess this in comparison to other studies?

b. Do you see advantages of this study design?

### Interview guide: Specialists on the molecular tumor board (Baseline)

#### Introduction

Thank you for taking a few minutes to speak with me.

As described in the email, alongside conducting the clinical study of routine ctDNA measurements, we are also investigating how specialists utilize the additional information for decision making.

I would like to ask you about:

How you plan to incorporate ctDNA analysis results into your recommendations.

How often you expect to reach different conclusions than without ctDNA analysis.

Whether you expect differences depending on the patient group / cancer diagnosis.

If you agree, I would like to record our conversation so I do not need to take notes. The transcripts will be anonymized so that you cannot be identified.

Do you have any questions before I start recording our conversation?

#### Questions

Could you explain your role on the molecular tumor board?

How do you plan to incorporate the additional information from ctDNA analysis into the tumor board's recommendations?

Based on which sources of information (e.g., databases, case studies) will you interpret the results to issue clinical recommendations?

Do you expect that the information from ctDNA will frequently lead you to make different recommendations than without this information? Why?

At what point in the disease progression do you expect an impact from ctDNA?

Do you anticipate differences in the utility of ctDNA among different patient groups / cancer diagnoses?

If yes, in which groups do you expect a particularly high benefit?

In which groups do you foresee lesser or no benefit?

### Interview guide: Pathologist(s) conducting ctDNA Analysis (Baseline)

#### Introduction

Thank you for taking a few minutes to speak with me.

As described in the email, we are attempting to qualitatively investigate key processes in the clinical study of the implementation of routine ctDNA measurements. We have identified ctDNA analysis in your molecular pathology as such a key process.

I would like to ask you about:

Anticipated challenges in ctDNA analysis.

Collaboration with oncology colleagues.

If you agree, I would like to record our conversation so I do not need to take notes. The transcripts will be anonymized so that you cannot be identified.

Do you have any questions before I start recording our conversation?

#### Questions

Can you describe the infrastructure and resources necessary for ctDNA analysis?

Do you think your laboratory can manage the increased amount of ctDNA measurements during the study?

What challenges have you experienced in analyzing ctDNA samples in the past and how have you handled them?

Do you anticipate new challenges for the LIQPLAT trial.

Can you explain specific protocols or methods you use to ensure high quality in ctDNA analysis?

How do you plan to communicate uncertainties or limitations associated with ctDNA analysis results to the clinical team?

Can you describe your collaboration with oncologists in integrating ctDNA analysis results into decisions for patient care?

Do you think this will change with now routine sample collections?

If yes, why?

### Interview Guide: Patients who were offered ctDNA

#### Introduction

Thank you for taking a few minutes to speak with me.

I would like to ask you some questions regarding the additional diagnostics that Dr. [X] has just offered you. Dr. [X] has suggested taking an additional tube of blood during your usual blood draw to measure the DNA (the genetic material) of the tumor.

I would like to ask you about:

Your experience of the first consultation with us.

How you experienced the offer of additional diagnostics.

What motivated you to accept or decline the additional diagnostics.

Your expectations of the additional diagnostics.

If you agree, I would like to record our conversation so I do not need to take notes. The transcripts will be anonymized so that you cannot be identified.

Do you have any questions before I start recording our conversation?

#### Questions

Why did you decide to participate in the study that was just offered to you, or why not?

How did you experience the offer of additional diagnostics as part of the study?

Did you feel well informed?

Did you feel overwhelmed with the decision?

What are your hopes for participating in the study / the additional analysis of the tumor DNA in your blood?

Do you have any concerns about participating in the study / the additional analysis of the tumor DNA in your blood?

If yes, what are they?

Would you like to add anything else?
