# Supplementary material for: Mixed-methods process evaluation of ctDNA use to guide decision-making in patients with advanced solid cancers: study protocol for a substudy of the LIQPLAT trial
Source: BMJ Open. 2025 Oct 28;15(10):e100537. doi: 10.1136/bmjopen-2025-100537 (PMC12570924; doi:10.1136/bmjopen-2025-100537)
Supplement: Supplementary file 3 [file bmjopen-2025-100537.reviewer_comments.pdf]

PEER REVIEW HISTORY

BMJ Open publishes all reviews undertaken for accepted manuscripts. Reviewers are asked to complete a checklist review form and are provided with free text boxes to elaborate on their assessment. These free text comments are reproduced below.

ARTICLE DETAILS

Title (Provisional)

A mixed-methods process evaluation of ctDNA use to guide decision-making in patients with advanced solid cancers: study protocol for a sub-study of the LIQPLAT trial

Authors

Schwenke, Johannes Manteo; Schmitt, Andreas M.; McLennan, Stuart; Janiaud, Perrine; Läubli, Heinz; Binder, Mascha; Alborelli, Ilaria; Matter, Matthias S.; Hinke, Jennifer; Widmer, Corinne C.; Hemkens, Lars G.; Kasenda, Benjamin; Briel, Matthias

---

VERSION 1 - REVIEW

**Reviewer**	**1**
**Name**	**Patelli, Giorgio**
**Affiliation**	**Università degli Studi di Milano**
**Date**	**08-Sep-2025**
**COI**	**None**

This study proposal by Schwenke and colleagues aims to evaluate the feasibility of ctDNA analyses in routine oncology practice at a single-center institution. The topic is certainly timely and of interest.

However, in its current form, the manuscript remains somewhat vague and could benefit from greater clarity. While the authors outline several objectives (acceptance rates, success rates, contribution to decision-making, attitudes of stakeholders), the absence of a clear statistical plan makes it difficult to see how these aims can be robustly addressed. As such, the study may currently lack the methodological rigor needed to ensure that the findings will be broadly interpretable.

Since the study has already received IRB approval, I am not sure to what extent modifications are feasible, but I believe it would strengthen the work if the authors explicitly

acknowledged this issue as a key limitation, in addition to the more minor limitations already listed.

VERSION 1 - AUTHOR RESPONSE

Reviewer: 1

Dr. Giorgio Patelli, Università degli Studi di Milano

Comments to the Author:

This study proposal by Schwenke and colleagues aims to evaluate the feasibility of ctDNA analyses in routine oncology practice at a single-center institution. The topic is certainly timely and of interest.

> We thank the reviewer for his valuable feedback.

1) However, in its current form, the manuscript remains somewhat vague and could benefit from greater clarity. While the authors outline several objectives (acceptance rates, success rates, contribution to decision-making, attitudes of stakeholders), the absence of a clear statistical plan makes it difficult to see how these aims can be robustly addressed.

> We thank the reviewer for this suggestion. We agree with the reviewer that prespecified statistical analysis plans can increase the robustness of research findings. We have updated the statistical analysis section to provide more detail on our methods of choice and referenced a version controlled statistical analysis plan on GitHub based on dummy data (https://github.com/scjohannes/liqplat-stat/blob/main/process-evluation-sap.pdf). We have fully rewritten the quantative analysis section as follows:
>
> > We will use a Bayesian framework to quantify uncertainty for all estimates, presenting results as posterior medians with 95% credible intervals. To quantify process indicators and explore the intervention's potential impact (see ***Error! Reference source not found.***), we will use Bayesian regression models, including logistic, negative binomial, and survival models as appropriate. This will allow us to estimate key metrics such as patient eligibility and acceptance rates, as well as the technical success and detection rates of the ctDNA workflow. Additionally, we will compare delivery of targeted therapies, time to treatment modification, and blood product transfusion rates between the intervention group and a registry-based external control group.
> >
> > For the Normalisation MeAsure Development (NoMAD) questionnaires, we will calculate descriptive statistics for each construct at each time point and visualize changes in scores over time. A detailed, version-controlled statistical analysis plan is publicly available on GitHub [17].

We would also like to emphasize that most findings of this process evaluation (concretely objectives 3-5), such as attitudes of stakeholders or *how* ctDNA influences decision making of the molecular tumour board are *qualitative* in nature and thus not subject to statistical analysis. It is therefore unclear to us how the lack of a prespecified statistical plan affects the robustness of these findings.

2) As such, the study may currently lack the methodological rigor needed to ensure that the findings will be broadly interpretable. Since the study has already received IRB approval, I am not sure to what extent modifications are feasible, but I believe it would strengthen the work if the authors explicitly acknowledged this issue as a key limitation, in addition to the more minor limitations already listed.

The detailed statistical analysis plan, of which any changes can be inspected because of version control, should address this concern.

Reviewer: 1

If you have selected 'Yes' above, please provide details of any competing interests.: Not applicable.
